# Automatic classification of IgA endomysial antibody test for celiac disease: a new method deploying machine learning

**DOI:** 10.1038/s41598-019-45679-x

**Published:** 2019-06-25

**Authors:** Florentino Luciano Caetano dos Santos, Irmina Maria Michalek, Kaija Laurila, Katri Kaukinen, Jari Hyttinen, Katri Lindfors

**Affiliations:** 1 Celiac Disease Research Center, Faculty of Medicine and Health Technology, Tampere University, Tampere, Finland; 2 Faculty of Social Sciences, Tampere University, Tampere, Finland; 3 Department of Internal Medicine, Tampere University Hospital, Tampere, Finland; 4 Computational Biophysics and Imaging Group, Faculty of Medicine and Health Technology, Tampere University, Tampere, Finland

**Keywords:** Diagnostic markers, Coeliac disease

## Abstract

Widespread use of endomysial autoantibody (EmA) test in diagnostics of celiac disease is limited due to its subjectivity and its requirement of an expert evaluator. The study aimed to determine whether machine learning can be applied to create a new observer-independent method of automatic assessment and classification of the EmA test for celiac disease. The study material comprised of 2597 high-quality IgA-class EmA images collected in 2017–2018. According to standard procedure, highly-experienced professional classified samples into the following four classes: I - positive, II - negative, III - IgA deficient, and IV - equivocal. Machine learning was deployed to create a classification model. The sensitivity and specificity of the model were 82.84% and 99.40%, respectively. The accuracy was 96.80%. The classification error was 3.20%. The area under the curve was 99.67%, 99.61%, 100%, and 99.89%, for I, II, III, and IV class, respectively. The mean assessment time per image was 16.11 seconds. This is the first study deploying machine learning for the automatic classification of IgA-class EmA test for celiac disease. The results indicate that using machine learning enables quick and precise EmA test analysis that can be further developed to simplify EmA analysis.

## Introduction

Celiac disease is an immune-mediated enteropathy driven by ingestion of gluten-containing cereals. The clinical course of the disorder is highly variable, and the gluten-induced symptoms may be both gastrointestinal as well as extra-intestinal. The spectrum of the above symptoms can range from mild to severe. Some patients may remain asymptomatic^1^. Such heterogeneity of clinical presentation makes diagnosing celiac disease challenging, and therefore, the disorder remains heavily unrecognized and underdiagnosed worldwide^2^.

Hitherto demonstration of small bowel mucosal villous atrophy, intraepithelial lymphocytosis, and crypt hyperplasia in biopsies obtained upon esophagogastroduodenoscopy has been the cornerstone for the diagnosis of celiac disease. Presence of celiac disease-specific IgA-class autoantibodies, determined by endomysial (EmA) and transglutaminase 2 autoantibody (TG2-Ab) assays, supports the diagnosis and serves as a valuable tool in selecting patients for endoscopy^3,4^. Presence of TG2-Ab in blood samples is detected using immunoassays enzyme-linked immunosorbent assay (ELISA) while the EmA test is an immunofluorescence-based method. The EmA test is regarded as the gold standard when determining the celiac disease autoantibodies, but due to its labor-intensiveness, high cost, and subjective interpretation, its use is limited in clinical practice^3^.

The value of celiac disease-specific autoantibody tests in the diagnostics is systematically acknowledged due to several limitations of the biopsy-based diagnosis, for instance, patchiness of small bowel mucosal lesions, difficulties regarding sampling, processing, and pathomorphological interpretation^5–15^. Moreover, the current celiac disease-specific antibody tests are highly accurate. Both EmA and TG2-Ab tests have excellent sensitivity (90–100%) and specificity (close to 100%)^1^. According to the current European Society for Pediatric Gastroenterology, Hepatology, and Nutrition (ESPGHAN) guidelines, the diagnosis of celiac disease can be made without a biopsy in symptomatic children with TG2-Abs exceeding 10 times the upper limit of normal in two independent measurements, positive EmA test, and celiac-type human leukocyte antigen haplotypes^6,16^. Recently, such diagnostic approach has been shown to be applicable also in adults^17^. It highlights the future importance of EmA testing and calls for solutions related to its labor-intensiveness and subjectivity.

The aim of this research was to determine whether supervised machine learning can be applied to create an automated method with expert comparable precision for the assessment and classification of the IgA-class EmA test for celiac disease.

## Results

**Figure 1:**
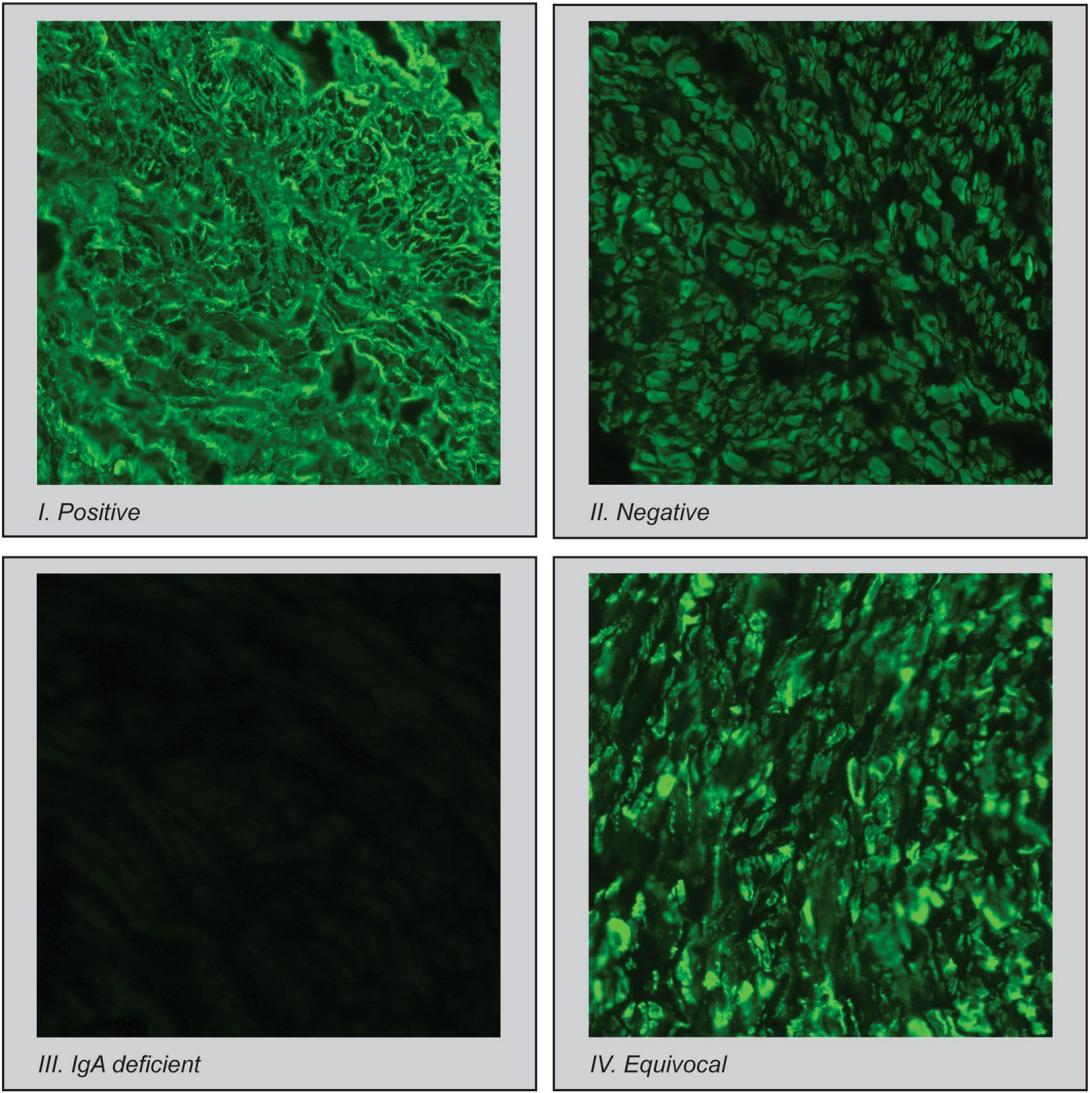
IgA-class EmA test classes.

In 2017-2018, in celiac disease service laboratory at the Tampere University, Tampere, Finland, 2597 high-quality images of IgA-class EmA test samples were obtained. All of them were classified as positive, negative, IgA deficient, or equivocal (Fig. 1) by an expert evaluator as presented in Table 1 and included in the study.

**Table 1:** Description of the dataset.

Class		Number of samples	Percent of the total dataset
I	Positive	274	10.55%
II	Negative	2260	87.02%
III	IgA deficient	13	0.50%
IV	Equivocal	50	1.93%

Machine learning was deployed to create a new method of automatic assessment and classification of the IgA-class EmA test for celiac disease. The classification was based in AdaBoost with SVM, and the sample features obtained through multi-scale, rotational invariant, co-occurrence among adjacent local binary patterns. Two SVM models were trained - Model 1 based on the whole sample-size (n = 2597) and supplemental Model 2 based on the randomly under-sampled size.

**Figure 2:**
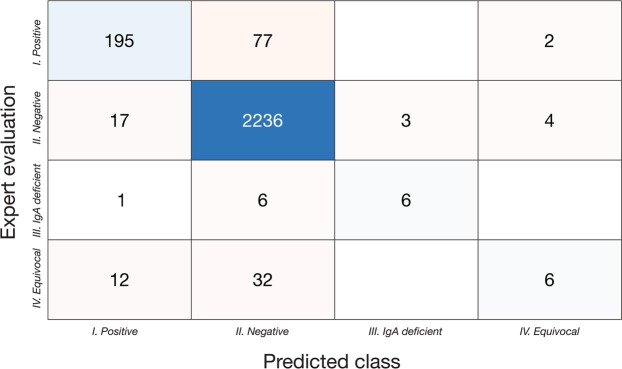
Confusion matrix of the classification model.

From 274 samples graded as positive by the expert evaluator, Model 1 predicted 195 to be positive, 77 negative, and 2 equivocal (Fig. 2). From 2260 samples graded as negative by the expert evaluator, the algorithm predicted 17 to be positive, 2236 negative, 3 as IgA deficient, and 4 as equivocal. Out of 13 samples classified by the expert as IgA deficient, 6 were classified by the algorithm as IgA deficient, 1 as positive, and 6 as negative. Finally, from the 50 equivocal samples defined by the expert evaluator, 12 were predicted to be positive, 32 negative, and 6 as equivocal.

**Figure 3:**
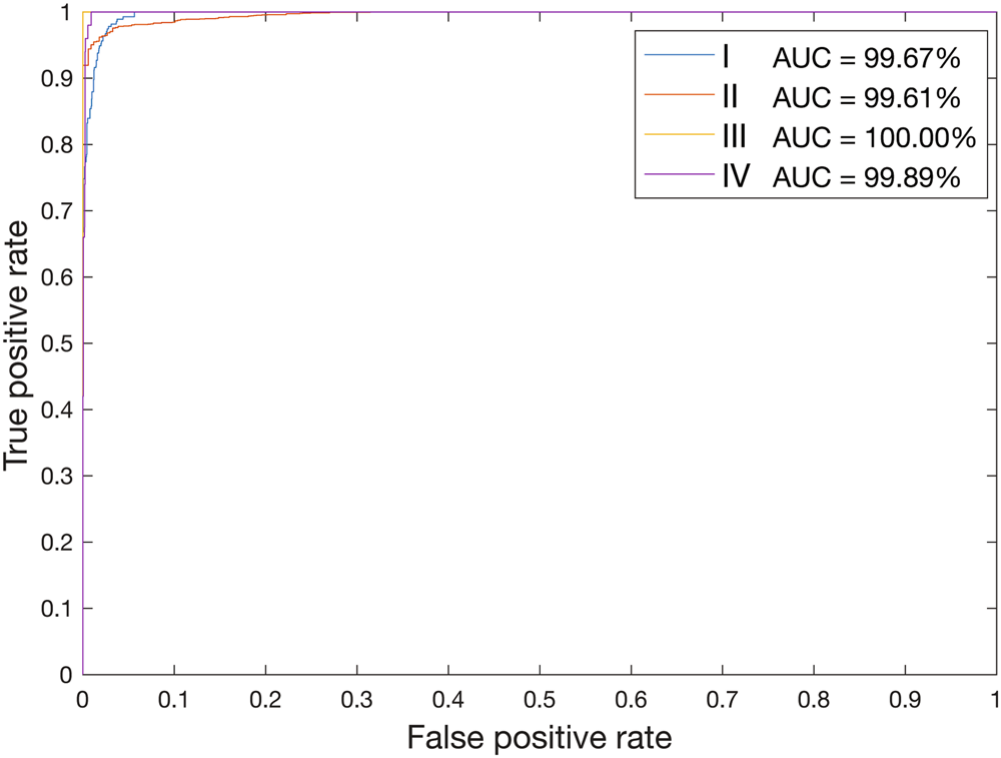
Receiver operating characteristic curves for the classification model.

The Model 1 developed in this research was characterized by accuracy of 96.80% and classification error of 3.20% (Table 2). The sensitivity was 82.84% and specificity 99.40%. The F_1_ score was 0.65 and Cohen’s kappa coefficient was 0.85. The AUC was 99.67%, 99.61%, 100%, and 99.89%, for I, II, III, and IV class, respectively (Fig. 3). The mean assessment time per image was 16.11 seconds (standard deviation of 0.52 seconds).

**Table 2:** Analysis of performance of Model 1 (based on the whole sample size) and Model 2 (supplemental, randomly under-sampled).

Measure of performance	Model 1	Model 2
Accuracy	96.80%	98.85%
Classification error	3.20%	1.15%
Sensitivity	82.84%	98.91%
Specificity	99.40%	98.81%
F_1_ score	0.65	0.75
Cohen’s kappa coefficient	0.85	0.98

To control for the possibility of multi-class classification problems supplemental Model 2 was created, deploying random under-sampling. The measures of performance of Model 2 were remarkably similar to the ones obtained with Model 1 (Table 2). The Model’s 2 accuracy was 98.85%, and the error rate was 1.15%. Its sensitivity and specificity were 98.91% and 98.81%, respectively. The F_1_ score was 0.75 and Cohen’s kappa coefficient was 0.98.

## Discussion

### Deployed machine learning methods

In this study, we selected SVMs for the development of automatic classification method for IgA-class EmA test, as they represent a class of non-probabilistic binary linear classifiers, able to map their inputs into high-dimensional feature spaces. They are a popular machine learning technique that has been applied successfully in other fields of biomedicine^18–24^. In this study, a feature descriptor, called multiscale co-occurrence among adjacent local binary patterns after edge enhancement, was applied. The descriptor is multi-scale, which means it is able to cope with different sizes of samples’ structures such as different vessels’ diameter, reticulin fibers’ length, and Wharton’s jelly conformation. Moreover, the descriptor is rotation invariant, allowing for a precise classification independently of the slides’ orientation in the microscope. Besides, it has a high descriptive power, which enables detecting subtle differences between samples and consequently their correct classification. The power of the descriptor results from the auto-correlation matrix generated by the multiple local binary patterns mapping. Such mapping enables adapting to non-uniform illumination conditions. Finally, due to the expansion of the canonical local binary pattern premise of center-based texture description to the inter-pattern correlation, the descriptor is dependent only on the contrast of magnitude between the central pattern and its surrounding patterns^25^.

The high variability between the number of samples in the four different groups might seem the main limitation of the study. However, when applying machine learning to clinical sciences, where usually classes are not represented equally, imbalanced data is a typical problem. Nevertheless, it does not mean that machine learning cannot be applied to clinical problems. In the case of the presented Model 1, one might expect to encounter multi-class classification problems. Datasets like ours, that represent a cross-section of subjects suspected for celiac disease, are typically imbalanced, which reflects the sample distribution in a genuine clinical setting. The vast majority of the patients will be in the “Negative” class and a very small minority will be in the “Positive” class.

To address the possibility of multi-class classification problems we used several techniques. First of all, AdaBoost ensemble technique was used to combine weak learners to create a strong learner that can make accurate predictions. AdaBoost deals with the class imbalance problem by maintaining a set of weights on the training dataset in the learning process. Moreover, 10-fold cross-validation provides positive class samples with a higher chance of inclusion in the training and testing phases. Furthermore, while presenting the results not only accuracy was showed, but also different performance metrics that are regarded as more correct in case of imbalanced datasets, i.e. a confusion matrix and ROC curves. Finally, a supplementary Model 2 was created, that deployed a sample obtained after random under-sampling without replacement.

In the case of the “IgA-deficient” class, representing only 0.50% of the dataset, overfitting was observed (AUC = 100%). However, as the model’s performance is reported by the AUC, which is computed according to a one-versus-all approach for multi-class problems, the obtained results are independent between classes. Possible future expansion of the presented research would be by increasing the samples’ number and balancing the samples distribution between classes.

### Limitations and strengths of the study

The developed method is dependent on the quality of the images that compose the dataset, which is one of the most common issues in the image-based machine learning field. Therefore, imaging parameters’ standardization and acquisition area that characterized the used dataset were crucial for the correct classification of the samples.

The principal advantage of the study is the large sample size (n = 2597 samples) that was used to create the classification model which represents the actual distribution of the EmA samples in clinical practice. Furthermore, the model was trained with the results of the evaluation made beforehand by a highly qualified specialist with over 20 years of experience in the assessment of IgA-class EmA test for celiac disease. Although only one expert graded the EmA images, this follows a quality-controlled standard procedure fulfilling European guidelines. Thus, the results show that machine learning can be used to provide similar precision as expert evaluation.

### Significance of the findings and suggestions for future research

The presented findings’ combination provides support for the conceptual premise that deploying machine learning for automatic classification of IgA-class EmA test in celiac disease is possible. These results suggest that creating a model characterized by both satisfying sensitivity and specificity is conceivable. To the knowledge of the authors, the method is the first fully-automated, user-independent algorithm deploying machine learning for IgA-class EmA test for celiac disease assessment published in the literature.

This study lays the groundwork for future research into the application of artificial intelligence modalities in the field of celiac disease diagnostics. Its empirical findings demonstrate that implementation of machine learning enables quick and precise EmA test analysis comparable to an expert-conducted evaluation and provides an automated method in the diagnostic work-up in addition to common TG2-Ab ELISA test. This approach could be extended by deploying bigger sample size and multiple expert sample evaluators.

The presented findings have important implications for expanding the use of IgA-EmA tests by healthcare professionals for celiac disease diagnostics. The use of machine learning to provide automatic analysis of IgA-EmA tests can facilitate work both in centers with a low incidence of celiac disease and in low-resource settings with little access to specialists able to provide a high-quality evaluation. Furthermore, deployment of machine learning algorithms would make the IgA-EmA test assessment less time-consuming and more cost-effective.

## Conclusions

In conclusion, in this study, a new method of the automatic analysis of IgA-class EmA test for celiac disease deploying machine learning was presented. It is the first report on using machine learning for automatic classification of IgA-class EmA tests for celiac disease. The model is characterized by the sensitivity of 82.84% and specificity of 99.40%. The mean assessment time is 16.11 seconds per sample. The study confirmed the possibility of machine learning application for automatic evaluation and classification of the IgA-class EmA test for celiac disease.

## Materials and Methods

### Study material

The study material included consecutive serum samples collected due to clinical suspicion of celiac disease and analyzed for EmA during the 2017-2018 period in celiac disease service laboratory at the Tampere University, Tampere, Finland. The laboratory serves as a reference entity and takes part in regular international external quality-control assessment by the United Kingdom National External Quality Assessment Scheme. The EmA test was standardized according to European standardization working group (ESPGHAN and European Medical Research Councils Clinical Network for Gastroenterological Immunology: Serological Screening for Celiac Disease)^26^.

The EmA testing was part of clinical studies approved by the Regional Ethics Committee of Tampere University Hospital District. In the above-mentioned studies, all study participants provided written informed consent.

### Determination of IgA-class EmA

The IgA-class EmA testing was carried out according to the protocol developed by the European Working Group on Serological Screening for Celiac Disease^26^. Five μm thick, full transverse human umbilical cord cryostat sections were placed on Vectabond^TM^ (Vector Laboratories, Burlingame, CA, USA)-pretreated slides. Serum samples were initially diluted 1:5 in phosphate-buffered saline (PBS, pH 7.4). The slides were incubated for 30 minutes with diluted serum samples at room temperature. Afterwards, they were washed in PBS (pH 7.4) and incubated with fluorescein isothiocyanate-conjugated rabbit F(ab’)_2_ anti-human IgA (dilution 1:160, DAKO A/S, Glostrup, Denmark) for 30 minutes at room temperature. After subsequent washing and mounting, the slides were analyzed with a fluorescence microscope (Olympus BX60, UPLANFL 20x objective). Positivity was defined as fluorescence of reticulin fibers in the vascular wall, external border of the vascular wall, and structures of Wharton’s jelly. All positive and equivocal sera were further diluted up to 1:4000 (1:5, 1:50, 1:100, 1:200, 1:500, 1:1000, 1:4000).

Following a quality-controlled standard procedure, all of the slides were classified by a specialist, with more than 20 years of experience in evaluation of IgA-class EmA, into one of the following four classes: I - positive, II - negative, III - IgA deficient, and IV - equivocal (Fig. 1).

The samples were photographed with pre-set camera settings (gamma 1.08, saturation 0.20, blue and red channels were excluded, green channel 7.04, acquisition time 1.00 s). The images were exported in.jpg format. To create the algorithm, a dataset of 2597 high-quality IgA-class EmA images was used. The above number of samples is the result of the collection of data on the diagnosis of patients with suspected celiac disease in a university hospital during the period of 22 months.

### Development of a classification model

**Figure 4:**
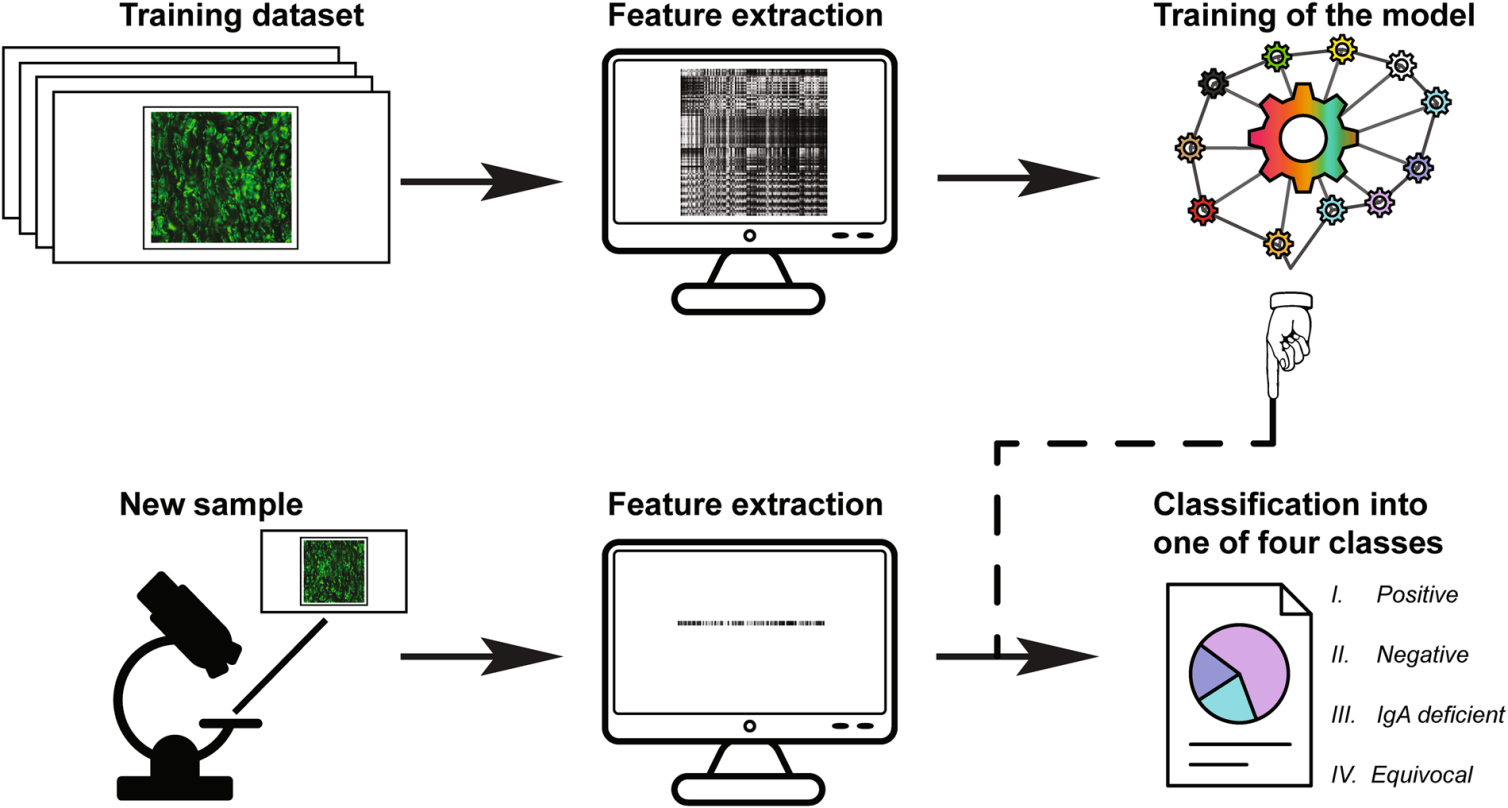
Development and usage of the classification model.

Conventionally, development of a classification model is a three-stage process (Fig. 4). During the first stage features of the images are extracted using feature descriptors. Next, the model is trained and tested. Finally, the performance of the created model is evaluated.

A feature descriptor proposed by Nosaka *et al*.^25^ was applied consecutively to all of the *a priori* blinded images from the dataset, to extract the feature set. After enhancing edges, the descriptor extracts the co-occurrence among adjacent local binary patterns with three search radiuses 1, 2, and 4 pixels. These provide multi-scale rotation invariance and high descriptive ability.

The previously prepared feature set was used to train error-correcting output codes multi-class support vector machine model (SVM) (with a radial basis function kernel, as a one-versus-all classifier, ten-fold cross-validated). In other words, given labeled training data, the SVM model, where a data point is viewed as a p-dimensional vector, was used to separate such points with (p-1) dimensional hyperplane and provide an optimal hyperplane which categorizes new examples.

In each training-testing fold, the dataset was divided randomly as following: 70% for training and 30% for testing. Every time, a one-versus-all SVM was trained for each class to prevent overfitting even in cases where the length of the feature vector was larger than the number of observations. To prevent overfitting, SVMs use regularization. It is done by applying non-linear kernels and tuning of the kernels and regularization parameters. Both tuning and regularization parameters are optimized by multiple (in this study 10-fold) and consecutive cross-validated training-and-testing. This decreases the generalization error, dependent on the margin (distance between class centers) but independent of the feature space^27^.

After mapping the feature vector into a complex hyperspace, to minimize exponential loss, the parameters were tuned using AdaBoost, a machine learning meta-algorithm resistant to overfitting^28^, with one hundred consecutive learning cycles. AdaBoost is based on a cycle of consecutive training and tuning of weak trainers’ set. After each iteration the weak trainers and their weights are tweaked to optimize the separation between classes. The weak trainers that misclassified a sample are discarded and replaced by new ones, with random parameters. Through such an evolution of many generations of weak learners, AdaBoost provides a classification method less prone to overfitting^29^. To control for ascertainment bias, decision trees (weak trainer models) with ten surrogate splits at each branch node were applied in the present methodology. With each AdaBoost iteration, the branches of the decision trees were pruned, and the weights recalculated, improving the classification performance.

In this study, two SVM models were trained. Model 1 was based on the whole sample size (n = 2597). Supplemental Model 2 was created to adjust the class distribution of a dataset and address the possibility of multi-class classification problems. Considering the dataset’s characteristics, Model 2 was created deploying random under-sampling. Samples from the majority class (negative) were randomly removed without replacement until the number of samples in negative class and the positive class (according to expert evaluation) was even.

Both of the classification models were developed and tested using MATLAB® (version R2018b, *Image Processing Toolbox, Statistics and Machine Learning Toolbox*).

### Model evaluation

To evaluate the performance of the models a confusion matrix was created, and the following parameters were calculated: classification error (using 10-fold cross-validation), sensitivity, specificity, accuracy, and the receiver operating characteristic curves for the calculation of the area under the curve (AUC). The performance-related information of a classifier was obtained creating a *classperformance* object by using the *classperf* function (MATLAB®; version R2018b). The accuracy, which defines the closeness of a measured value to an expert-evaluation value, is a positive scalar defined as the number of correctly classified samples divided by the number of classified samples, where inconclusive results are not counted^30^. The error rate of the classifier is a positive scalar, defined as the number of incorrectly classified samples divided by the number of classified samples^30^. The sensitivity of the classifier is as a positive scalar, defined as the number of correctly classified positive samples divided by the number of true positive samples^30^. The specificity of the classifier is a positive scalar, defined as the number of correctly classified negative samples divided by the number of true negative samples^30^. F_1_ score is a positive scalar, defined as the harmonic average of precision (samples correctly classified as positive out of all positive classified samples) and sensitivity^31^. Cohen’s kappa coefficient is a positive scalar defined as the quotient of the difference between the agreement rate and the hypothetical probability of chance agreement, and the complement of the hypothetical probability of chance agreement^32^. The AUCs were obtained deploying the *perfcurve* function (MATLAB®; version R2018b). Finally, mean analysis time per image was calculated.

## Data Availability

The datasets generated and/or analyzed during the current study are available from the corresponding author on reasonable request and subject to the ethical approvals in place and material transfer agreements.
